# Community based sociotherapy for depressive symptomatology of Congolese refugees in Rwanda and Uganda (CoSTAR): a protocol for a cluster randomised controlled trial

**DOI:** 10.1080/20008066.2022.2151281

**Published:** 2023-01-20

**Authors:** Daniel M. Kagabo, Paul Bangirana, Girvan Burnside, Anna Chiumento, Rui Duarte, Darius Gishoma, Michelle Girvan, Angela Jansen, Stefan Jansen, Rosco Kasujja, Rachel Lubunga, Sarah Nevitt, Lucie Nzaramba, Emmanuel Sarabwe, Clare Jackson, Atif Rahman, Annemiek Richters, Jude Robinson, Theoneste Rutayisire, Peter Ventevogel, Ross G. White

**Affiliations:** a Mental Health & Community Psychology and Behaviour Research Group, College of Medicine and Health Sciences, University of Rwanda, Kigali, Rwanda; b Department of Psychiatry, Makerere University, College of Health Sciences, Kampala, Uganda; c Department of Health Data Science, University of Liverpool, Liverpool, UK; d Community Based Sociotherapy Rwanda, Kigali, Rwanda; e Center for Mental Health, College of Medicine and Health Sciences, University of Rwanda, Kigali, Rwanda; f Department of Mental Health and Community Psychology, Makerere University, Kampala, Uganda; g Department of Primary Care and Mental Health, University of Liverpool, Liverpool, UK; h Institute of Health and Wellbeing, University of Glasgow, Glasgow, UK; i Division of Resilience and Solutions, Public Health Section, United Nations High Commissioner for Refugees, Geneva, Switzerland

**Keywords:** Refugees, psychosocial, mental health, depression, sociotherapy, trial protocol, Refugiados, psicosocial, salud mental, depresión, socioterapia, protocolo de ensayo, 难民, 社会心理, 心理健康, 抑郁, 社会治疗, 试验方案

## Abstract

**Background:** Conflict in the Democratic Republic of Congo has led to large numbers of refugees fleeing to Uganda and Rwanda. Refugees experience elevated levels of adverse events and daily stressors, which are associated with common mental health difficulties such as depression. The current cluster randomised controlled trial aims to investigate whether an adapted form of Community-based Sociotherapy (aCBS) is effective and cost-effective in reducing depressive symptomatology experienced by Congolese refugees in Uganda and Rwanda.

**Methods:** A two-arm, single-blind cluster randomised controlled trial (cRCT) will be conducted in Kyangwali settlement, Uganda and Gihembe camp, Rwanda. Sixty-four clusters will be recruited and randomly assigned to either aCBS or Enhanced Care As Usual (ECAU). aCBS, a 15-session group-based intervention, will be facilitated by two people drawn from the refugee communities. The primary outcome measure will be self-reported levels of depressive symptomatology (PHQ-9) at 18-weeks post-randomisation. Secondary outcomes will include levels of mental health difficulties, subjective wellbeing, post-displacement stress, perceived social support, social capital, quality of life, and PTSD symptoms at 18-week and 32-week post-randomisation. Cost effectiveness of aCBS will be measured in terms of health care costs (cost per Disability Adjusted Life Year, DALY) compared to ECAU. A process evaluation will be undertaken to investigate the implementation of aCBS.

**Conclusion**: This cRCT will be the first investigating aCBS for mental health difficulties experienced by refugees and will contribute to knowledge about the use of psychosocial interventions for refugees at a time when levels of forced migration are at a record high.

**Trial registration:** ISRCTN.org identifier: ISRCTN20474555.

## HIGHLIGHTS

- There is a need to evaluate community-based psychosocial interventions for refugees.
- Community-based sociotherapy has been used to support communities in post-conflict situations but has not been evaluated in a randomised controlled trial.
- This protocol outlines a proposed randomised controlled trial of community-based sociotherapy adapted for Congolese refugees in Uganda and Rwanda.

## Abstract (es)

**Antecedentes:** El conflicto en la República Democrática del Congo ha llevado a un gran número de refugiados a huir a Uganda y Ruanda. Los refugiados experimentan niveles elevados de eventos adversos y factores estresantes diarios, que se asocian con problemas de salud mental comunes, como la depresión. El ensayo controlado aleatorio grupal actual tiene como objetivo investigar si una forma adaptada de socioterapia basada en la comunidad (aCBS en su sigla en inglés) es efectiva y rentable para reducir la sintomatología depresiva experimentada por los refugiados congoleños en Uganda y Ruanda.

**Método:** Se llevará a cabo un ensayo controlado aleatorizado por conglomerados simple ciego (cRCT, por sus siglas en inglés) de dos brazos en el asentamiento de Kyangwali, Uganda y el campamento de Gihembe, Ruanda. Sesenta y cuatro grupos serán reclutados y asignados aleatoriamente a aCBS o a la condición de cuidado usual mejorado (ECAU en su sigla en inglés). aCBS, una intervención grupal de 15 sesiones, será facilitada por dos personas provenientes de las comunidades de refugiados. La medida de resultado primaria serán los niveles autoinformados de sintomatología depresiva (PHQ-9) a las 18 semanas posteriores a la aleatorización. Los resultados secundarios incluirán niveles de dificultades de salud mental, bienestar subjetivo, estrés posterior al desplazamiento, apoyo social percibido, capital social, calidad de vida y síntomas de TEPT a las 18 y 32 semanas posteriores a la aleatorización. La rentabilidad de aCBS se medirá en términos de costos de atención médica (costo por año de vida ajustado por discapacidad, DALY en su sigla en inglés) en comparación con ECAU. Se llevará a cabo una evaluación del proceso para investigar la implementación de aCBS.

**Conclusión:** Este cRCT será el primero en investigar aCBS para las dificultades de salud mental que experimentan los refugiados y contribuirá al conocimiento sobre el uso de intervenciones psicosociales para refugiados en un momento en que los niveles de migración forzada están en un nivel récord.

## Abstract (zh)

**背景：**刚果民主共和国的冲突导致大量难民逃往乌干达和卢旺达。难民经历了更高水平的不良事件和日常应激源，这与抑郁等常见心理健康问题有关。当前的集群随机对照试验旨在考查一种改编形式的社区社会疗法 (aCBS) 在减少乌干达和卢旺达的刚果难民所体验到的抑郁症状方面是否有效且具有成本效益。

**方法：**将在乌干达康瓦利定居点和卢旺达吉亨贝营地进行双臂、单盲集群随机对照试验 (cRCT)。将招募 64个集群并随机分配给 aCBS 或加强日常护理 (ECAU)。 aCBS 是一项 15 个疗程的团体干预，将由来自难民社区的两名人员引导。主要结果指标将是随机分组后 18 周时自我报告的抑郁症状水平 (PHQ-9)。次要结果将包括随机分组后 18 周和 32 周的心理健康困难水平、主观幸福感、流离失所后应激、社会支持感、社会资本、生活质量和 PTSD 症状。与 ECAU 相比，aCBS 的成本效益将根据医疗保健成本（每个残疾调节生命年的成本，DALY）来衡量。将进行过程评估以考查 aCBS 的实施情况。

**结论：**本 cRCT 将是第一个针对难民所经历心理健康困难 aCBS的考查，将有助于了解在被迫移民水平达到历史最高水平时对难民使用社会心理干预的知识。

**试验注册：**ISRCTN20474555

## 1. Background

Across the world, increasing numbers of people are being impacted by humanitarian crises and armed conflicts, with currently 84 million people forcibly displaced from their homes (UNHCR, 2021a). Most displaced populations are hosted in low- and middle-income countries (LMIC) where they often face protracted periods of displacement (UNHCR, 2021b). Countries in Sub-Saharan Africa alone are projected to host more than 31 million forcibly displaced people – approximately 40% of all forcibly displaced people (UNHCR, 2022). Approximately six million people have been forcibly displaced from their homes in the Democratic Republic of the Congo (DRC), a low-income country in central Africa that has experienced three major conflicts in the last 20 years (UNHCR, 2020). In terms of refugees fleeing DRC, Uganda (421,563) and Rwanda (74,491) are the first and fourth largest recipients (UNHCR, 2021c).

Experiences of conflict and forced displacement are associated with the development of mental health difficulties. An estimated one in five conflict-affected people (including internally displaced people and refugees) experience a mental disorder, which is about double that of non-affected populations (Charlson et al., 2019). Whilst an increased risk of experiencing trauma is an important consideration, Turrini et al.’s (2017) umbrella review of prevalence rates of common mental disorders experienced by refugees and asylum seekers found that rates of depression and anxiety were as high as rates of post-traumatic stress disorder. Important drivers of the increase in mental health issues in forcibly displaced populations are: (1) experiences of violence associated with conflict and oppression, and (2) displacement-related stressors such as: lack of access to basic resources and livelihoods, lack of safety and security, family violence, community tensions, social isolation and break-down of supportive social systems (Miller & Rasmussen, 2010; Silove et al., 2017; White & Van der Boor, 2021). Low levels of social cohesion within communities have been highlighted as a strong predictor of depressive symptomatology (Helbich et al., 2020). Furthermore, the mental health and psychosocial well-being of Congolese refugees in Rwanda and Uganda have been shown to be negatively impacted by low levels of social cohesion in family and community relations (Chiumento et al., 2020; Ingabire & Richters, 2020). Previous research conducted in the DRC has indicated that interventions, such as cognitive processing therapy, can improve aspect of people’s social situation including structured social capital (Hall et al., 2014).

Within the rapidly evolving field of Mental health and Psychosocial Support (MHPSS), it is therefore a priority to develop more evidence around community-based psychosocial methods for common mental health difficulties that focus on social connectedness and interpersonal ‘healing’ (Jones & Ventevogel, 2021). One such psychosocial method is community-based sociotherapy (CBS; Dekker, 2018), an intensive lay-facilitator-led group intervention, in which participants, over the course of 15 sessions, go through a phased process that aims to: restore and strengthen feelings of safety, trust, and dignity; and to promote social cohesion and mutual care in communities affected by violent conflicts or natural disasters. The intervention was developed and successfully scaled up in Rwanda (Richters et al., 2008; Richters et al., 2010; Scholte et al., 2011), but has not yet been evaluated in a refugee context or using a randomised controlled trial design. No research to date has investigated whether CBS, with its focus on social determinants of mental health, such as social cohesion and mutual support, can reduce elevated levels of depressive symptomatology.

### 1.1. Aims and objectives

The Community-based Sociotherapy Adapted for Refugee Settings (CoSTAR) project aims to conduct a cluster randomised controlled trial (cRCT) to evaluate if an adapted form of Community Based Sociotherapy (aCBS) intervention is more effective in reducing levels of depressive symptomatology experienced by Congolese refugees in Rwanda and Uganda than enhanced care as usual (ECAU). Secondary aims include investigating whether CBS is cost-effective and what factors need to be considered in bringing CBS to scale in humanitarian settings.

### 1.2. Study hypotheses

1. aCBS intervention will be significantly superior to ECAU in lowering the levels of depressive symptomatology experienced by Congolese refugees at 18-weeks (primary endpoint).
2. aCBS will be significantly superior to ECAU in reducing levels of depressive symptomatology of Congolese refugees at 32-weeks, and at increasing 18- and 32-week secondary outcomes levels of wellbeing and health-related quality of life.
3. Congolese refugees in the aCBS intervention arm will incur significantly lower health care costs (cost per DALY) compared with the ECAU group at 18-weeks and 32-weeks follow up.

In the trial, the minimum clinically important difference on the primary outcome measure will be considered to be 2-points. A justification for this is provided later in the paper.

## 2. Methods

### 2.1. Design

**Figure 1.**
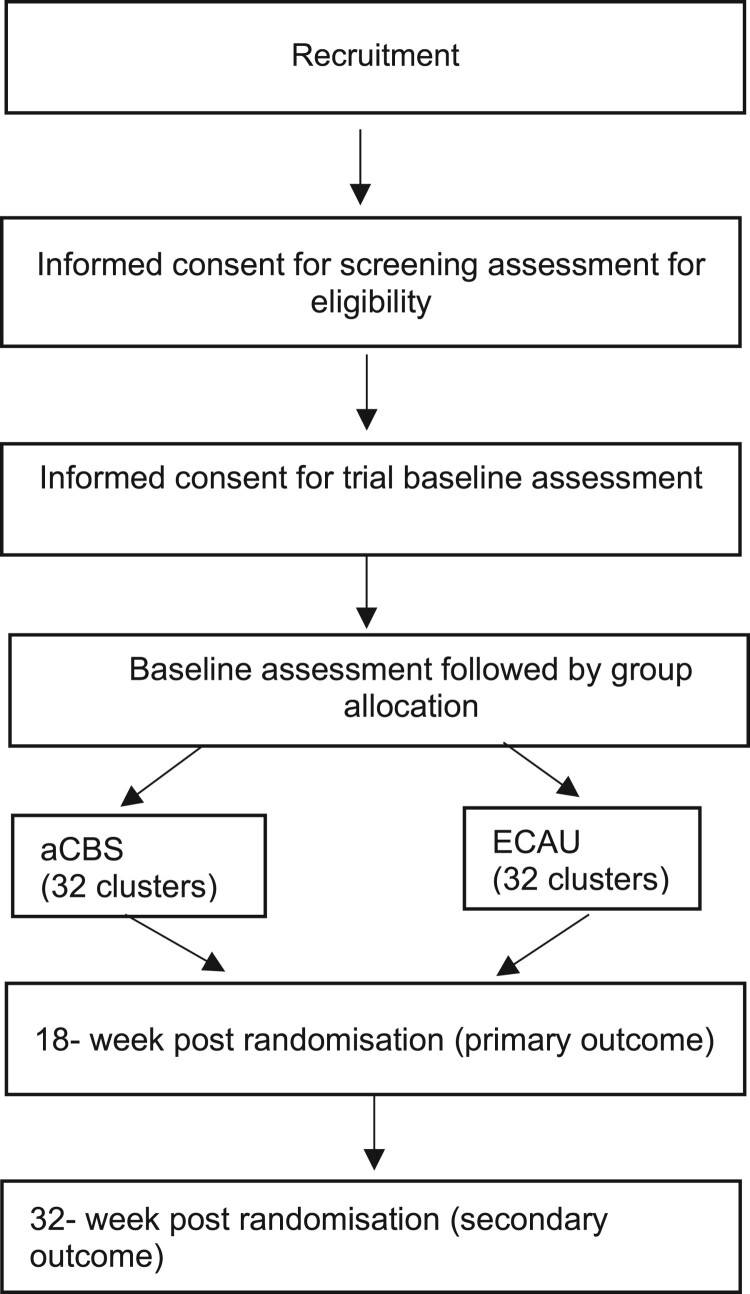
cRCT Flow Diagram.

We will use a two-arm cRCT to linguistically and culturally adapt, implement, and evaluate a community-based psychosocial intervention in two Congolese refugee settings in Uganda and Rwanda (ISRCTN Registry (2019) Number: ISRCTN20474555). The first phase of the CoSTAR project was an exploratory formative phase aimed at developing an understanding of the difficulties the refugee populations face, and adapting assessment instruments for use in both study sites accordingly – for further details see Chiumento et al. (2020), Robinson et al. (2022), and Kasujja et al. (2022). The second phase will comprise the definitive trial phases, nested economic evaluation, and an embedded qualitative process evaluation. Our approach to the current project has been informed by the Updated UK Medical Research Council Guidance: *A new Framework for the Developing and Evaluating Complex Interventions* (Skivington et al., 2021). Clusters (i.e. villages in the Rwandan refugee camp and blocks in the Ugandan refugee settlement) will be allocated 1:1 to either a CBS or ECAU. Participants will be assessed at baseline before group allocation, and at 18-and 32-weeks post-randomisation. The cRCT will be reported according to the CONSORT statement and details of study participation presented in a CONSORT flow diagram (see Figure 1) (Eldridge et al., 2016; Zwarenstein et al., 2008).

### 2.2. Changes to trial design due to COVID-19 impact

The COVID-19 pandemic led to changes to our planned cRCT design (see the CONSERVE-SPIRIT checklist by Orkin et al., 2021 in the Appendix). Research activity in a planned internal pilot had to cease in March 2020. Subsequently, adaptations to the trial design were made in conjunction with the joint Trial Steering Committee and Data Safety Management Committee (TSC/DSMC) and the trial sponsor (University of Liverpool). Based on the recruitment rate, the decision was taken to progress to the definitive trial and to regard the internal pilot phase as an external pilot instead. Members of the CoSTAR research team and facilitators of the group sessions (both aCBS and ECAU) are adhering to the social distancing and personal hygiene guidance aimed at restricting the spread of COVID-19 issued by the national governments in the recruitment sites in Uganda and Rwanda. A standard operating procedure detailing guidelines for each site was circulated and associated training delivered by respective site coordinators.

### 2.3. Participants recruitment, consent, and screening

The recruitment procedure for the trial participants will follow the ‘door to door’ approach used previously in a refugee camp to recruit participants to an evaluation of the *Self-Help Plus* intervention in Uganda (Brown et al., 2018; Tol et al., 2020). This consistent, systematic approach will be used to identify and approach households to screen for potential participants. Within households, potential eligible people (Table 1) will be approached by the Research Assistant (RA) followed by the two step consenting procedures. If more than one adult meets the eligibility criteria in a household, the RA will randomly select a person by drawing numbered slips of paper and inviting the person who drew the slip numbered as ‘one’ to participate. Informed consent will be sought for eligibility screening, and those who are eligible invited to provide informed consent for participation in the cRCT. During this procedure, the RA will explain the objectives of the CBS intervention and verify that the potential participants are aware that they will be offered the opportunity to attend group sessions. The participants will be provided with a copy of the Participant Information Sheet (PIS) to consider whether they wish to participate. If they decline, the RA will proceed to the next household. The RA will continue this process until a target number of 15 eligible people within that particular cluster have been identified and consented into the research project. The RA will then repeat the procedure in another cluster in the camp. For those who provide informed consent to participate in the trial, the baseline assessments will be administered by an RA within one week.

**Table 1.** Inclusion and exclusion criteria.

Inclusion criteria	Exclusion criteria
Identifies as a Congolese	Self-reported current diagnosis of a complex mental disorder in response to a screening question (e.g. psychotic disorders, post-traumatic stress disorder (PTSD), substance dependence) **
Age 18 years and above	Self-reported severe cognitive impairment in response to a screening question (e.g. severe intellectual disability, dementia)
Residing within the Gihembe refugee camp, Rwanda or Kyangwali settlement, Uganda	Self-reported as participating or being enrolled in a study of a psychosocial or psychological intervention at the time of recruitment
Have a self-reported good level of fluency in the languages that aCBS will be delivered in (Kinyarwanda in Rwanda / Kiswahili in Uganda)	Self-reported active suicidal intent in response to screening question**
Willing to participate in group meetings	

** Individuals that are excluded because of a diagnosis of a mental disorder or imminent risk of suicide will be referred for urgent local mental health support.

### 2.4. Setting

In Rwanda, the Gihembe Refugee Camp is located in the Northern province and is one of the oldest Congolese refugee camps and home to 12,341 refugees from the DRC as of January 2021. The camp was established in December 1997 to host survivors of the massacre in Mudende camp, another camp for Congolese refugees in Rwanda. The main language spoken is Congolese Kinyarwanda (UNHCR, Rwanda, 2021d).

In Uganda, the Kyangwali settlement in the Western province was established in the 1960s to accommodate refugees from Rwanda. After the 1994 voluntary repatriation of Rwandan refugees, it predominantly hosts refugees from the DRC. As of January 2021, the settlement was home to 125,039 persons with the majority (96%) of refugees from the DRC. The main language spoken is Congolese Swahili (UNHCR, Uganda, 2021e).

CoSTAR engages a diverse range of stakeholders including representatives from refugee communities from the two camps, local academic institutions, the UNHCR and Non-Government Organisations (NGOs) including Community Protection and Health implementing partners. The Office of the Prime Minister in Uganda and the Ministry of Emergency Management in Rwanda, the government regulatory bodies assigned to emergency situations, will provide site access and facilitation of the CoSTAR cRCT, together with UNHCR.

### 2.5. Intervention: community-based sociotherapy

Community-based Sociotherapy (CBS) is an intervention that aims to strengthen agency and harnesses existing community resources to foster social cohesion, meaningful social relations and mutual trust as part of peacebuilding among conflict-affected groups or populations. Groups of between 10–15 people engage in facilitated discussions, participate in exercises and games, sing songs and engage in other forms of cultural expressions aimed at addressing issues that can include ongoing daily stress, past experiences and threats to safety and trust within people’s communities. CBS is purposively inclusive as an intervention – it brings together a cross-section of adults of different genders and ages living in a specific geographic area (Jansen et al., 2015). Ordinarily, CBS is implemented in post-conflict settings using a community-detection approach whereby community members who may be presenting with psychosocial issues can be identified and invited by the community-based sociotherapists to attend weekly sessions. CBS has been described as an approach that uses ‘the interactions between individuals and their social environment to facilitate the re-establishment of values, norms, and relationships and at the same time provides participants the opportunity for debate, the sharing of experiences and coping mechanisms’ (WHO & CGF, 2014, 33).

CBS sessions are delivered by two trained lay facilitators recruited from within the population of interest. Over the course of 15 weekly group sessions of 3-hours duration participants are taken through a process of six phases i.e. safety, trust, care, respect, say in rule-making, and processing emotions (Dekker, 2018). Further details about the intervention have been described in a handbook by Cora Dekker (Dekker, 2018). The *United Nations High Commissioner for Refugees* has noted the potential benefit of CBS for refugees (UNHCR, 2021f). Drawing on CBS Rwanda’s 16 years of experience delivering CBS in Rwanda, an adequate dose of CBS will be regarded as 11 or more sessions out of the total 15.

The CBS intervention has been linguistically and contextually adapted for use in Congolese refugee settings. Supervision of facilitators will be coordinated by CBS Rwanda. All training materials and resources have been developed by CBS Rwanda. The fidelity of aCBS delivery will be monitored in a subset (10%) of randomly selected sessions using a checklist developed for the conduct of the cRCT. Those involved in the aCBS arm will remain eligible to receive routine care according to conventional practice and following local regulations in the refugee camp setting.

### 2.6. Enhanced care as usual (ECAU)

As with the CBS arm, the ECAU arm of the cRCT will involve participants receiving routine care according to conventional practice and following local regulations in the refugee camp setting. This care as usual will be enhanced by delivering training to NGO staff and healthcare workers in the *Inter-Agency Standing Committee* (IASC) Guidelines on Mental health and Psychosocial support in Emergency Settings (IASC, 2007), and the reviewing/updating of mental health referral pathways in the study that were gathered in Phase 1 of the CoSTAR Project activity. To control for the group meeting format of aCBS, the 10–15 participants recruited in clusters randomised to ECAU will be invited to attend weekly group meetings for a period of 15 weeks. The group sessions, running for 1-2 h, will be facilitated by lay-persons recruited from the refugee community in conjunction with members of the Refugee Community Advisory Group (RCAG) and will focus on (1) community updates/announcements, and (2) educational material relating to *UN Sustainable Development Goals*. All materials developed for group sessions will be standardised across the settings.

### 2.7. Participants withdrawal and dropout

A *Withdrawal Form* will be completed when a group member communicates that he or she does not want to continue participating in all trial related activities including follow-up assessments. Attendance rate to group sessions in either the aCBS or ECAU arm will be monitored. *Dropout Forms* will be completed if a participant wishes to stop participating in group sessions. The *Withdrawal Forms* and *Dropout Forms* will be completed by the aCBS or ECAU group facilitator, and a copy shared with the research team. All participants will be followed up for assessments unless he or she has completed a *Withdrawl Form* and does not want to continue involvement in the cRCT.

### 2.8. Outcome measures

Assessments will be completed at baseline, 18-week post-randomisation, and 32-week post-randomisation. Table 2 provides a schedule for the assessments. The primary outcome will be levels of self-reported depressive symptomatology (PHQ-9) at 18 weeks post-baseline. The Mini International Neuropsychiatric Interview (Sheehan et al., 1998) has been included at baseline to help demonstrate criterion validity for the PHQ-9. Secondary outcomes will include levels of subjective wellbeing, post-displacement stress, perceived social support, social capital, quality of life, traumatic life events, and levels of PTSD symptoms at 18-week and 32-week post-randomisation. We have adapted assessment instruments for use in both sites (Kasujja et al., 2022). Outcome assessors will be blinded to group allocation. Owing to the COVID-19 outbreak which began prior to the commencement of the definitive trial, a COVID-19 questionnaire containing 6 items for assessing participants’ level of concerns about COVID-19 was added to the assessment tools. This will be administered at each of the three assessment points.

**Table 2.** Schedule of assessments.

Outcome measures				Administration time point		
				Baseline Week 0	Primary outcome Week 18	Second outcome Week 32
Assessment Tool		Concept	Citation			
						
*Screening for eligibility*						
Screening form		Eligibility		Y	N	N
Mental capacity form		Mental Capacity		Y	Y	Y
						
*Consenting for participation*						
PIS and Informed consent				Y	N	N
Demographic Form		Sociodemographic information		Y	N	N
Primary outcome						
*Patient Health Questionnaire*	PHQ 9	Levels of depressive symptoms	Kroenke et al., 2001	Y	Y	Y*
Secondary outcomes						
*Self-Reporting Questionnaire*	SRQ 20	Levels of common mental disorders	Scholte et al., 2011	Y	Y	Y
*Mini-International Neuropsychiatric Interview (The Depressive Episodes Module and Module ‘O’)*	MINI	Levels of depressive episodes & ruling out organic causes of disorders	Sheehan et al., 1998	Y	N	N
*Checklist for Daily Life Stressors*	CDES	Post-displacement stress	Riley et al., 2017	Y	Y	Y
*World Health Organization (five) Wellbeing Index*	WHO-5	Subjective well-being	WHO, 1998	Y	Y	Y
*Multi-dimensional Scale of Perceived Social Support*	MSPSS	Perception of social support	Canty-Mitchell & Zimet, 2000, Zimet et al., 1988	Y	Y	Y
*Shortened and Adapted Social Capital Assessment Tool*	SASCAT	Social capital	De Silva et al., 2006	Y	Y	Y
*World Health Organization Quality of Life BREF*	WHOQOL	Quality of Life	WHOQOL Group, 1998	Y	Y	Y
*PTSD Checklist – Screener*	PCL-6	Screen Post Traumatic Disorder	Lang & Stein, 2005	Y	Y	Y
*Trauma Events Inventory (derived from the Harvard Trauma Questionnaire – Part I)*		Screen for Traumatic Events	Riley et al., 2017	Y	N	N
*Adapted Client Service Receipt Inventory*	CSRI	Health resource use	Beecham & Knapp, 1992	N	Y	Y
COVID 19 Questions		Level of concerns about coronavirus		Y	Y	Y
Group session monitoring forms		Group session data		Completed by facilitators at the end of each CBS session		
Adverse event form		Adverse events		(Completed as required)		

Y = Yes, N = No.

*PHQ-9 score at the 32-week time-point will be assessed as a secondary outcome.

### 2.9. Health economic evaluation

The economic evaluation aims to estimate the cost effectiveness of the aCBS intervention when compared to ECAU to reduce the depressive symptomatology of Congolese refugees in Gihembe camp and Kyangwali settlement over the duration of the cRCT. It is expected that four categories of resource use, and the corresponding costs, will be considered in the economic evaluation: intervention costs, healthcare costs, prescribed medications and over the counter (OTC) medications, and productivity costs. The effect measure for the analysis will be disability-adjusted life years (DALYs).

The CSRI (Beecham & Knapp, 1992) will be adapted for use with a refugee population in Uganda and Rwanda and will be the main resource use collection tool. Modifications to the CSRI were initially discussed during the project launch with representatives of Community Based Sociotherapy (CBS) Rwanda, Humanitarian Initiative Just Relief Aid (HIJRA) Uganda and the Ministry of Emergency Management (MINEMA) Rwanda. The CSRI was piloted in two aCBS groups (*n* = 26) and two ECAU groups (*n* = 30) during the discontinued pilot trial. CSRI completion took between 10–15 min with 100% (56/56) response rate. Healthcare resource utilisation will be collected for each participant at the follow-up points (i.e. 18 and 32-weeks post-randomisation) using the adapted CSRI. For the outpatient resource use, information on the number of visits, purpose of visit, duration of visit and staff designation (e.g. nurse, pharmacist, mental healthcare worker, etc.) will be collected. The per-hour unit cost by staff designation will be obtained from national sources or published literature. For hospital admission, cost will be calculated as a function of primary indication for admission (e.g. malaria, pneumonia, etc.), type of service provider (charity, private or public) and duration of the admission episode. Country-specific and hospital-specific unit cost estimates for secondary and tertiary services in Uganda and Rwanda will be obtained from the WHO-CHOICE project (WHO, 2021). Unit costs for medications will be obtained from the International Drug Price Indicator Guide (MSH 2015). Productivity cost will be calculated by multiplying the income lost per day by the number of forgone workdays both of which are collected in the CSRI. If the workdays forgone is reported and the income per day is not reported, then productivity cost will be obtained by multiplying the forgone workdays by the median national income for the reported type of work. All resource use items, unit costs and sources will be reported. Cost components will be added up to derive total client level costs. The unit costs of services in both Rwanda and Uganda will be translated to a common currency (US Dollars) by use of purchasing power parities reported by the International Monetary Fund (IMF, 2020).

Analyses will be reported in accord with Consolidated Health Economic Evaluation Reporting Standards (CHEERS) recommendations (Husereau et al., 2013). A within-trial cost consequence analysis will be carried out to estimate mean resource utilisation, costs and total DALYs in each group, together with relevant measures of sampling uncertainty (e.g. mean, standard deviation (SD), median). Mean differences between groups will be presented with 95% confidence intervals. The economic evaluation will take the form of a cost-effectiveness analysis, to calculate the cost per DALY averted. Base-case analyses will be conducted from a health sector perspective, with additional analyses presented from a societal perspective (i.e. absence from work by the client, family member or friend due to the client's health condition).

The primary outcome for the economic evaluation will be the incremental cost-effectiveness ratio (ICER), defined as the incremental cost of aCBS when compared to ECAU per additional DALY averted.

### 2.10. Sample size and power calculation

A minimal clinically important difference (MCID) of 5 points on the PHQ-9 for people with depression has previously been identified – with an estimated SD of 5.8 (Löwe et al., 2004). Research with a large sample of refugees living in camps in sub-Saharan Africa suggests that approximately 40% of the sample will meet criteria for elevated depressive symptoms (PHQ-9 > 5) (Feyera et al., 2015). Our recent surveying (paper in preparation) of the refugee communities in Gihembe and Kyangwali (*n* = 359), which has used the same recruitment strategy to be used in the current trial, indicated that 35% of the people surveyed had moderate to severe levels of depressive symptomatology, and that the mean PHQ-9 score for the sample was 7.6 (SD = 5.5). As less variability in change is expected in those with low baseline levels of depressive symptoms recruited to the cRCT, a SD of 6.0 will be assumed as a conservative overestimate. As it is estimated that 65% of the sample will not have elevated levels of depressive symptoms at baseline, a reduced MCID of 2 points on the PHQ-9 will be assumed for the current cRCT. In one of the few studies that has empirically investigated the matter, Kounali et al. (2022) concluded that a clinically important difference on the PHQ-9 (indexed against the *Global Rating of Change Scale*) corresponds to a 21% (95% confidence interval (CI) −26.7 to −14.9) reduction from baseline PHQ-9 scores. Our choice of a 2 point MCID on the PHQ-9 is more than 21% of the mean baseline score obtained in our earlier community survey. Based on an average cluster size of 10 participants, to achieve 80% statistical power at a 5% (2-sided) statistical significance level, with a conservative intra-cluster correlation coefficient of 0.1, we would require 30 clusters per arm. We aim to recruit at least 64 clusters (32 per arm) to allow for potential attrition (6%) (Vos et al., 2012).

### 2.11. Changes to trial design due to relocation of refugees from gihembe site

In March 2021, it was announced that four clusters recruited to the definitive cRCT in Gihembe, Rwanda were part of a scheme to relocate members of the Congolese refugee communities to Mahama refugee camp in Rwanda. Due to logistical and budgetary constraints these clusters will be lost to follow-up. To ensure that the definitive trial is adequately powered for the planned analyses, the decision was taken with the TSC/DSMC and Sponsor to increase the planned number of clusters recruited in Kyangwali, Uganda from 32 to 42 (it was not possible to increase the number of clusters recruited in Gihembe due to the limited number of available clusters). Thus, the total number of clusters to be recruited into the definitive trial across both sites is 70 (*n* = 1050).

### 2.12. Randomisation

Clusters of participants in Gihembe camp (i.e. villages) and Kyangwali settlement (i.e. blocks of dwellings) will be randomised to aCBS or ECAU with a ratio of 1:1. Stratified block randomisation will be conducted by a statistician not otherwise involved in the cRCT using a web-based system developed by the Liverpool Clinical Trial Centre (LCTC), University of Liverpool. Randomisation will be done after the participants are recruited. The outcome of the randomisation will be communicated directly to the Trial Coordinator (based at University of Rwanda) who will ensure allocation concealment and will not release the randomisation code to the research team until commencement of analysis. An RA who was involved in recruiting, consenting, and conducting baseline assessments will return to participants within the cluster to inform them about the outcome of randomisation. For the clusters randomised to aCBS, the RA will be accompanied by a group facilitator who will be delivering the aCBS sessions. This encounter will provide an opportunity for early engagement with the aCBS group session attendees and opportunities to answer questions or address any concerns that they might have about the intervention. This adaptation aims to ensure ecological validity of the intervention delivery, as usually CBS participants would be recruited directly by facilitators. The RA accompanying an aCBS group facilitator to communicate allocation will not be involved in any follow up assessment in order to maintain allocation concealment.

### 2.13. Blinding of interventions

The nature of the aCBS intervention and ECAU control does not allow blinding of intervention participants and facilitators. Investigators collecting primary and secondary outcome data at 18- (primary endpoint) and 32-weeks (secondary endpoint) post-randomisation follow-up, and the statistical team performing all analyses, will be blinded to the allocation status of the participants.

In addition, to preserve blinding:

1. We will ask the members of the research team to avoid interaction with study participants outside the study setting.
2. Local RAs involved in the assessments will be instructed on how follow-up assessments should be conducted to preserve effective blinding and will remind participants and facilitators not to share information about any specific activities they have been participating in at the start of follow-up assessments.
3. We will ask NGOs to provide appropriate spaces for intervention delivery and private spaces, where possible, for the administration of assessments.
4. The primary outcome (PHQ-9) will be the first assessment administered, and the CSRI will be administered at the end of an assessment as this instrument has the potential to unblind the RA due to the nature of the questions asked.

### 2.14. Contamination of intervention

Although the use of clusters as the unit of randomisation serves to minimise the risk of contamination, there is a theoretical possibility of contamination by recruiting refugees who might interact with each other and therefore divulge intervention components from people in the experimental arm to those in the control condition. To minimise the possibility of any form of contamination, participants in both the aCBS and the ECAU cluster will be asked to refrain from sharing study-related information and materials during the study.

### 2.15. Trial management

The day-to-day management of the cRCT will be undertaken by the Trial Management Group (TMG) which will be chaired by the Chief Investigator (CI) and will include the Trial Coordinator, local PIs, co-investigators, and site coordinators. Monitoring the conduct of the cRCT and related activities (including the health economic evaluation and process evaluation) will be provided by the TSC/DSMC. The TSC/DSMC will include four independent experts whose responsibilities will include advising the TMG on any trial management, data management/analysis, ethics and safety monitoring. The cRCT will be sponsored by the University of Liverpool. A representative of the Sponsor will also attend the TSC/DSMC meetings. We will also convene a Project Oversight Group (POG) to meet on a 3-monthly basis, bringing together representatives from stakeholders involved in the cRCT – including UNHCR, implementing community protection and health partners, CBS Rwanda, and representatives from the RCAG.

### 2.16. Data management

A record identification (ID) will be created to assign a unique trial ID to participants. No personally identifiable data will be recorded on this log. Data will be stored in the secure cloud-based systems used by Research Electronic Data Capture (REDCap: https://www.project-redcap.org). Data entry into REDCap database will be performed by a delegated research team member. As per the delegation log, RAs involved in administering assessments will not be involved in data entry. Quantitative data in field sites will be collected using paper-based questionnaire booklets. The hard copies of the assessments will be stored securely inside a locked cabinet at a secured office at Makerere University, Uganda and University of Rwanda, Rwanda.

### 2.17. Statistical analysis

Analyses of the trial data will be on an intention-to-treat basis whereby all participants will be analysed according to the arm to which their cluster was randomised. To account for the clustering of individuals within refugee settings, mixed-modelling statistical methodology will be employed. Linear or non-linear regression methods, depending on distribution of the outcome variable, will be employed (i.e. via PROC MIXED, PROC GLIMMIX, or PROC NLMIXED within SAS Statistical Software version 9.4) for the analysis of primary and secondary outcomes. Potential effect modifiers, such as baseline characteristics (e.g. age, gender) and exposure to traumatic life events assessed at baseline will be included as covariates within the regression models. No formal interim analyses or analyses by treatment group of the accumulating data will be performed. The analysis of primary and secondary outcomes will only be commenced once the data set has been checked, cleaned and locked. These analyses will be conducted according to appropriate guidance (Aiken et al., 1991, Preacher et al., 2007). A full statistical analysis plan will be approved and signed off by the LCTC and by the TSC/DSMC prior to any comparative analyses being conducted.

### 2.18. Qualitative process evaluation

A qualitative process evaluation will be conducted to explore: facilitator and participant experiences of participating in the aCBS intervention or the ECAU groups; views of family members of those participating in the aCBS intervention; and community stakeholders’ views of the acceptability of aCBS within their community. These data will be obtained through semi-structured individual interviews (face-to-face or telephone depending on COVID-19 restrictions) and focus group discussions with purposively selected participants. Interviews and focus groups will explore topics including: the perceived accessibility, acceptability and ability of aCBS to address primary mental health and wellbeing concerns of refugees; how aCBS compares to or complements other forms of mental health, social and/or economic support that may be available; and experiences of trial participation and/or administration of research assessments. We will also seek to conduct ethnographically-informed observations of aCBS sessions to consider group dynamics and the approaches of aCBS facilitators and Theory of Change workshops to revisit and update pathways to impact from an initial theory of change developed at the formative research stage. All participants in this phase will be over 18 years of age and with full capacity to consent to participate in qualitative interviews or focus group discussions. Data from this process evaluation will be transcribed in the original language, and subsequently translated into English by bi-lingual RAs involved in the original discussions.

Taking an inductive approach, we will use thematic analysis (Green & Thorogood, 2018) to identify codes and themes in the data, referring to both the original language and English transcripts. The analysis will be led by members of the Process Evaluation research team, using NVivo software to assist with data management and coding. We will ensure rigour through continual discussion of the emerging findings to clarify key ideas and concepts across languages and sites. This approach recognises the methodological complexities of qualitative cross-language research (e.g. Chiumento et al., 2017; Temple, 1997; Temple & Young, 2004), and the importance of ongoing discussions to ensure that analysis remains situated within the lived-realities of participants, deferring to original language transcripts over the English translations. Adopting this approach seeks to move away from the hegemony of English as the language of research, foregrounding the expertise and interpretations of our qualitative RAs in the analysis and dissemination of our findings (Kohrt et al., 2014).

### 2.19. Ethical approvals and adverse events

Ethical approvals for all aspects of the project have been obtained from the University of Liverpool (ref: 4860), Makerere University (MAKSS REC 11.18.237) and the University of Rwanda (Ref: No 065/CMHS IRB/2019). Informed consent will be obtained prior to trial participation, and all data will be treated confidentially and stored securely according to LCTC standard operating procedures (SOP).

All defined sets of Reportable Serious Adverse Events (RSAEs) reported by participants or observed by group facilitators or research staff will be recorded. Safety reporting of related adverse events will occur during the study from the period of randomisation until the end of involvement in the definitive trial. It is recognised that the recruited population can be considered vulnerable as a result of past and ongoing adverse events and daily living stressors. As such, there may be elevated levels of mental health difficulties. A *Distress Protocol* and associated SOP has been developed to direct research staff in supporting individuals presenting as a risk to themselves and others, and/or at risk from harm for others at assessment points or during aCBS intervention sessions. Guided by the Distress Protocol, data on potential adverse event, action taken in response to the event, and the outcome of the adverse event will be entered on a specifically developed form. The assessment of the seriousness and relatedness to the trial of the adverse event will be made by the delegated clinically trained research team members in Rwanda and Uganda who have become aware of the event. All SAEs are to be communicated to the in-country Principal Investigator immediately and a report submitted to the Chief Investigator within 24 h. RSAEs will in turn be reported to the combined the TSC/DSMC who will decide the appropriate response (e.g. contacting ethics committees within fifteen days of receiving the report if the SAE breeches stopping rules due to intervention harms). The Sponsor will be informed of all SAEs.

## 3. Discussion

CBS is a non-diagnostic high-intensity group-based psychosocial intervention that purports to address factors operating across different levels of people’s social environments which may in turn impact on levels of depressive symptomatology. Many of these factors have been highlighted as important for the mental health and wellbeing of forcibly displaced people (Chiumento et al., 2020; Robinson et al., 2022; White & Van der Boor, 2021). Although, preliminary research evidence conducted in conflict affected countries (Richters et al., 2008; Richters et al., 2010; Scholte et al., 2011) attests to CBS’s acceptability and potential efficacy, this is the first cRCT that aims to evaluate whether CBS will be effective at reducing levels of depressive symptomatology experienced by refugee communities. Members of the refugee communities will be trained relatively quickly and efficiently to act as sociotherapists. Fidelity to the core content of the study is ensured through context adapted training materials, provision of session guides, and supportive supervision. This cRCT will also assess the cost-effectiveness of aCBS for reducing depressive symptomatology experienced by Congolese refugees in Rwanda and Uganda. If sufficient evidence is established, implementation guidance will be developed in conjunction with UNHCR to assist with the adaptation, implementation and evaluation of CBS in diverse settings.

## Data Availability

Data sharing is not applicable to this article as no new data were created or analyzed in this study.

## Appendix – CONSERVE-SPIRIT checklist.

**Table:**

CONSERVE-SPIRIT Extension: 20 October 2021					
Item	Item title	Description			Page No.
I.	Extenuating Circumstances	*Describe the circumstances and how they constitute extenuating circumstances*. - As a result of the COVID19 outbreak, research activity relating to COSTAR Project pilot cRCT ceased in Rwanda and Uganda on the 16 and 20 March 2020 respectively. In addition, the Sponsor (University of Liverpool) of the research announced on 20 March that all recruitment to clinical trials must temporarily stop until further notice. - The pausing of the trial activity in the two sites severely impacted on the integrity of the pilot trial. The vast majority of the clusters that had been randomised as part of the internal pilot had received less than half of the required 15 community-based sociotherapy sessions, or 15 inactive group meetings. In addition, it has not been possible to commence the 18-week post-randomisation follow-up assessments. As such, we have not had the opportunity to determine whether *Criterion 2* (relating to participant retention) of the ‘stop/go criteria’ has been met			
II.	Important Modifications	*a. Describe how the modifications are important modifications*. - The primary analysis for the trial is based on outcomes collected at 18 weeks post-randomisation. The absence of these outcomes for the pilot phase due to the disruption caused by the COVID19 outbreak means these clusters cannot contribute anything to the analysis. To address the predicament, we discussed the possibility of the internal pilot becoming an ‘external pilot’. The key difference being that the data collected during the pilot phase would now not be included in the trial analysis on the basis that COVID19-related disruption (to the delivery of the intervention and control sessions, inability to complete follow-up assessments etc.) has prevented a fair evaluation of the intervention during the pilot phase.			
		*b. Describe the impacts mitigating strategies, including their rationale and implications for the trial*. - The initial power calculation conducted prior to trial commencement had indicated that 72 clusters were required (24 of which were the internal pilot) to adequately power the trial statistical analysis. This was based on conservative estimates of relevant parameters. - Mitigations: We revisited the power calculation and determined that at least 60 clusters will provide sufficient power for the statistical analyses we planned. This is 25% more than the additional 48 clusters that we had planned to recruit if the pilot data had been included in the analyses. However, we believe that this additional work can be accommodated. We would aim to recruit 64 clusters using a steeper recruitment curve in the time remaining. - Implication: Inclusion of a section in the Participant Information Sheet on ethical conduct related to compliance with recommended COVID19 preventive measures by the Uganda and Rwanda Government. The COVID19 guideline that are reflective of respective national governments and research ethical committees were circulated to teams at trial sites and along with it; an associated training was delivered across trial sites. In addition, a COVI19 evaluation form containing 6 items to assess participants level of concerns about COVID19 was added to the assessment battery for the definitive trial.			(see below)
		*c. Provide a modification timeline*. - Date when extenuating circumstance was identified: March 2020 Date when the circumstances took effect: May 2020 Number of participants/clusters enrolled to trial before the extenuating circumstance: 24 clusters - Project timelines was extended to run through 31st March 2022. Previously project duration was 30 months set to run from 1 September 2019–28 February 2021.			
III.	Responsible Parties	*State who planned, reviewed and approved the modifications*. - Planned by: Principal Investigator and local PIs at the Trial Site - Reviewed and approved by: Members of the independent joint Trial Steering Committee/ Data Management and Ethics Committee (TSC/DMEC) and the funder (ESRC GCFR), and ethical committees from Liverpool University (Sponsor), University of Rwanda, Makerere University			
IV.	Interim data	*If modifications were informed by trial data, describe how the interim data were used, including whether they were examined by study group, and whether the individuals reviewing the data were blinded to the treatment allocation*. Not applicable.			
SPIRIT Item and Number		For each row, if important modifications occurred, check one or both of ‘impact’ and/or ‘mitigating strategy’ and describe the changes in the protocol. Check ‘no change’ for items that are unaffected in the extenuating circumstance.			Page No.
		No Change	Impact*	Mitigating Strategy**	
1	Title	✓			
2	Trial registration	✓			
3	Protocol version		✓	Version 13, dated 28 May 2020 changes on: - Trial sample size (i.e. number of clusters) - Trial design reconfiguration - Participant Information Sheet for Definitive Trial updated to include information on COVID19 preventive measures - Circulation and training on guideline on COVID19 preventive measure to promote adherence through end of study period Version 14, dated 15 September 2020. Addition of: - Six COVID19 related impact questions Version 16, dated 23 July 2021 - Increased data collection method options to include telephone interviews whenever face-to-face interviews not feasible.	
4	Funding	✓			
5	Roles and responsibilities	✓			
6	Background and rationale	✓			
7	Objectives	✓			
8	Trial design		✓	Permission was sought to abandon activity on the pilot trial and instead recruit 64 new clusters to undertake a fully powered definitive trial with no pilot phase’	
9	Study setting	✓			
10	Eligibility criteria	✓			
11	Interventions	✓			
12	Outcomes	✓			

13	Participant timeline		✓	Suspension and then resumption of activities at different point in the study duration affected the schedule of previously planned activities. We sought updated ethical approval for revised timelines/ procedures from all committees overseeing the project	
14	Sample size		✓	Prior to V13 of the protocol (May 2020) the plan had been to recruit 72 clusters (including 24 clusters recruited as part of an internal pilot). However due to COVID19 related disruption, permission was sought to abandon activity on the pilot trial and instead recruit 64 new clusters to undertake a fully powered definitive trial with no internal pilot phase. In March 2021, it was announced that four clusters recruited to the definitive cRCT in Gihembe, Rwanda were part of a scheme to relocate members of the Congolese refugee communities to Mahama refugee camp in Rwanda. These individuals were in the end lost to follow-up. To ensure that the definitive trial is adequately powered for the planned analyses, the number of clusters recruited in Kyangwali, Uganda were increased from 32 to 42. It was not possible to increase the number of clusters recruited in Gihembe due to the limited number of clusters available. This then brought the total number of clusters recruited in the definitive trial across both sites from 64 to 70. We sought updated ethical approval for from all committees overseeing the project	
15	Recruitment	✓			
16	Allocation	✓			
17	Blinding (masking)	✓			
18	Data collection methods		✓	For process evaluation, telephone was added to face-face as an options for data collection method from individual interviews. This was all depending on COVID-19 restriction. We sought updated ethical approval for from all committees overseeing the project	
19	Data management	✓			
20	Statistical methods	✓			
21	Data monitoring	✓			
22	Harms	✓			
23	Auditing	✓			
24	Research ethics approval		✓	For revised study design, revised timelines/ procedures we sought updated ethical approval for from all committees overseeing the project	
25	Protocol amendments		✓	Amendments relating to activities and schedule changes as a consequence to COVID-19 in-country guidelines were made and sought ethical approval for all revisions from all committees overseeing the project. We sought updated ethical approval for from all committees overseeing the project	
26	Consent or assent		✓		
27	Confidentiality	✓			
28	Declaration of interests	✓			
29	Access to data	✓			
30	Ancillary and post-trial care	✓			
31	Dissemination policy	✓			
32	Informed consent materials		✓	Addition of a paragraph to participant information sheet (PIS) compliance with measures aimed at minimising spread of COVID19 in order to protect research participants and the research team. We sought updated ethical approval for from all committees overseeing the project	
33	Biological specimens	✓			

*Aspects of the trial that are directly affected or changed by the extenuating circumstance and are not under the control of investigators, sponsor or funder.

**Aspects of the trial that are modified by the study investigators, sponsor or funder to respond to the extenuating circumstance or manage the direct impacts on the trial.

The CONSERVE-SPIRIT Checklist is licenced by the CONSERVE Group under the Creative Commons Attribution-NonCommercial-NoDerivs 4.0 International licence.
